# The Mechanical Properties of Polyurethane-Solidified Ballast with and Without Geogrid Reinforcement

**DOI:** 10.3390/ma19143099

**Published:** 2026-07-19

**Authors:** Jingshang Xiao, Shuojun Chen, Wei Chen, Xuanjun Wang, Xinyuan Liu, Zhiqing Liu, Minzhe Yu

**Affiliations:** 1 International College of Engineering, Changsha University of Science and Technology, Changsha 410114, China; 13873770501@163.com; 2 School of Civil Engineering, Central South University, Changsha 410075, China; cuckoo275@163.com (S.C.); 18390653009@163.com (X.L.); 244812289@csu.edu.cn (Z.L.); 244811165@csu.edu.cn (M.Y.)

**Keywords:** polyurethane-solidified ballast, geogrid, mechanical property, laboratory test, finite element simulation

## Abstract

Polyurethane-solidified ballast (PSB) technology and geogrid reinforcement can be used to strengthen ballast beds. However, there has been little research on the mechanical properties of ballast beds under the combined effects of these two methods. This study addresses this research gap through an integrated experimental–numerical investigation of the cooperative reinforcement mechanisms. Uniaxial compression and oblique shear tests were conducted on standard and geogrid-reinforced PSB specimens, and a three-dimensional random aggregate finite element model was developed to represent the actual ballast gradation. The results show that geogrid reinforcement increased the compressive strength by 42.1% and reduced the maximum lateral deformation by 14.2%, while having little influence on the elastic modulus and Poisson’s ratio. In the oblique shear tests, geogrid reinforcement increased the peak loads by 14.2–23.0% compared with the standard specimen. A directional dependence was also observed, with the highest peak load occurring when the shear plane corresponded to the unreinforced surface. These findings clarify the cooperative reinforcement mechanism between polyurethane solidification and geogrid confinement and provide a mechanical basis for designing reinforced ballasted trackbeds in heavy-haul railways subjected to high axle loads and pronounced lateral deformation, as well as in high-speed railway lines requiring enhanced track stability.

## 1. Introduction

The railway ballast bed forms the lower foundation of the track, serving primarily to support and transmit train loads. Based on structural differences, ballast beds are generally classified into two main categories: ballasted and ballastless, each with distinct characteristics in terms of construction methods and maintenance requirements. Ballasted tracks, owing to their low construction costs and ease of localized repairs and track geometry adjustments, are frequently used in railway projects with complex geological conditions. However, inherent issues with traditional ballasted tracks—such as the susceptibility of ballast to fragmentation and the tendency of the ballast bed to deform—increase the risk of track instability and raise railway maintenance costs [1,2]. Ballastless track consists of a monolithic subgrade formed by concrete structures, creating a continuous rigid bearing layer that provides high integrity and excellent stability; nevertheless, this also limits track adjustment capabilities, making it difficult to apply in areas with unstable subgrades, such as soft soil embankments or tunnels crossing faults [3,4]. Consequently, polyurethane-solidified track bed (PSTB) has emerged, combining the stability of ballastless track beds with the high adjustability of ballasted track beds [5], and offering a new solution for addressing issues related to unstable subgrades in railway engineering.

The core principle of PSTB involves mixing isocyanates, polyols, and catalysts to trigger a chemical reaction that produces a viscoelastic polymer capable of bonding the ballast [6]. Early applications resulted in rigid PSTB, where the stabilization mechanism relied primarily on contact points between consolidated particles, with the voids not fully filled. Through continuous development, flexible solidification technology now allows the polyurethane to fully foam and fill the interparticle voids, transforming the originally bulk ballast bed into a continuous, unified structural entity. This effectively restricts ballast displacement and stress transmission paths induced by train dynamic loads, delays the accumulation of residual deformation, slows the process of ballast fragmentation and degradation, and offers excellent vibration-damping and noise-reduction properties [7,8,9,10,11,12].

The addition of polyurethane fundamentally alters the contact state between ballast particles in traditional ballasted track beds and introduces complex tensile, shear, bending, and torsional forces into the track bed structure [13], prompting extensive research into the mechanical properties of PSB itself. Yu et al. [14] conducted an in-depth analysis of the compressive characteristics and deformation behavior of PSB cubic specimens under free-edge conditions, employing both uniaxial and cyclic loading test protocols. Keene et al. [15] investigated the changes in mechanical properties after polyurethane bonding through three-point bending tests, unconfined compressive strength tests, and cyclic triaxial compression tests, concluding that PSB exhibits good compressive strength, flexural strength, and elastic deformation capacity. Lee et al. [16] conducted large-scale triaxial tests on polyurethane-mixed coarse aggregates, quantifying a linear positive correlation between the stiffness and strength of PSB and the polyurethane content, which can be used to predict basic mechanical properties of polyurethane. Gundavaram and Hussaini [17] conducted large-scale direct shear tests on polyurethane-mixed ballast and found that polyurethane-bonded ballast exhibits higher shear strength than geogrid-reinforced ballast and superior resistance to particle fragmentation. Zhang et al. [18] developed a new laboratory testing method and found that the mechanical behavior of PSB is highly dependent on bonding thickness and specimen size. Prasad and Hussaini [19] determined through large-scale direct shear and cyclic loading tests that a polyurethane content of 2.25% is the optimal choice for balancing ballast service performance and economic efficiency, as it can significantly reduce ballast deformation and degradation and extend the track maintenance cycle. Liu et al. [20] conducted uniaxial compression tests on rigid polyurethane composite aggregates of different densities and proposed a three-stage constitutive model that accounts for specimen density, establishing a unified macro-micro understanding of compressive behavior.

In railway ballast reinforcement, geogrids are widely used to reinforce the ballast layer due to their mechanical interlocking mechanism with ballast particles, thereby limiting lateral particle displacement, reducing vertical settlement, and enhancing overall stability. Mishra et al. [21] noted that both square- and triangular-aperture geogrids can effectively increase the peak deviatoric stress of ballast specimens; however, the reinforcement effect is highly dependent on the matching relationship between aperture size and the average particle size ($D_{50}$) of the ballast. A reasonable aperture-to-particle-size ratio can maximize the particle-geogrid interlocking effect. Qian et al. [22] further compared the reinforcement effects of square, rectangular, and triangular aperture geogrids through large-scale triaxial shear tests and discrete element method (DEM) simulations, finding that triangular aperture geogrids performed best in reducing permanent deformation due to their ability to provide more uniform confinement resistance in all horizontal directions. Prasad and Hussaini [23] placed geogrids at the bottom of the ballast layer and injected elastic polyurethane into the top region of the ballast above the geogrid’s influence zone. utilizing a synergistic mechanism of “geogrid bottom anchoring + polyurethane top bonding.” This significantly reduced vertical settlement and lateral deformation while substantially improving the modulus of elasticity, damping ratio, and track stiffness. Indraratna et al. [24] found through large-scale cyclic loading tests that when $A/D_{50}$ > 0.95, optimal performance is achieved by placing the geogrid approximately 65–76 mm from the ballast-subgrade interface; whereas when $A/D_{50}$ < 0.95, placing it directly at the ballast-subgrade interface is more effective.

However, a critical theoretical gap is evident in all the above studies: existing work has treated polyurethane solidification (chemical bonding) and geogrid reinforcement (physical confinement) as two separate and independent technical routes, without addressing the coupling mechanisms that arise when both coexist in the same ballast bed system. Specifically, the filling of voids by polyurethane drastically changes the friction coefficient, overall stiffness, and load-transfer paths among ballast particles, which inevitably alters the interlocking boundary conditions and load-transfer efficiency between the geogrid and the ballast. Conversely, the external confinement introduced by the geogrid also significantly affects the stress redistribution and cracking evolution within the solidified ballast. This “chemical–physical” interaction has long been overlooked, resulting in the inability of current design theories to scientifically guide the engineering practice of composite-reinforced track beds, and a lack of quantitative predictive capability for the mechanical response under coupled effects.

To fill this research gap, this study is the first to systematically incorporate both polyurethane solidification and geogrid reinforcement into a unified experimental-numerical analysis framework. The core novelties are threefold: (1) Through comparative uniaxial compression and oblique shear tests on standard PSB specimens and geogrid-reinforced PSB specimens, we quantitatively reveal, for the first time, the influence of geogrid confinement on compressive strength, shear performance, and deformation modes of solidified ballast—particularly uncovering a remarkable directional dependence of shear strength with respect to the shear plane position, an unconventional phenomenon never reported in previous independent studies; (2) We introduce an advanced random aggregate modeling technique to construct a three-dimensional mesoscale finite element model of PSB that for the first time accounts for actual gradation distribution, overcoming the limitations of conventional homogenization assumptions and enabling refined numerical reproduction of the complex contact behavior among polyurethane, ballast, and geogrid; (3) Through bidirectional calibration and validation between experiments and simulations, we establish a parameterized model capable of accurately predicting the mechanical response of composite track beds, providing a theoretical basis for optimizing geogrid placement strategies and polyurethane dosage.

## 2. Laboratory Tests of Polyurethane-Solidified Ballast

### 2.1. Preparation of the Specimens

#### 2.1.1. Bulk Ballast

**Figure 1:**
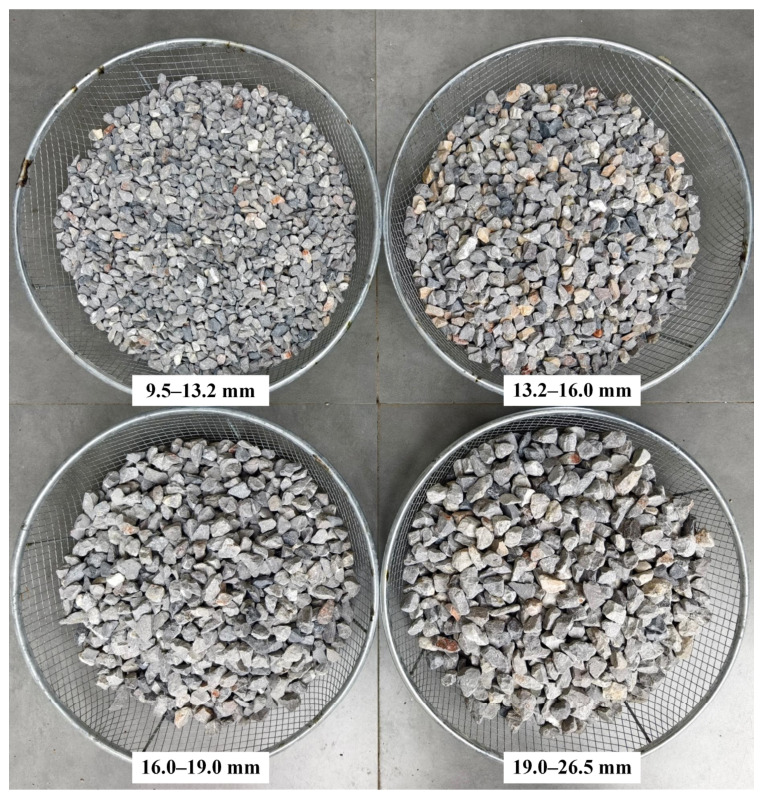
The four types of aggregate with different particle size ranges obtained after sieving.

Based on previous studies [25,26], this experiment selected 150 mm cubic specimens. The ballast aggregate used in this experiment was provided by Hunan Junjia Road Surface Materials Co., Ltd. (Changsha, China). To meet the required gradation standards, the ballast aggregate was sieved using square-mesh sieves, yielding four different particle size ranges: 26.5 mm–19.0 mm, 19.0 mm–16.0 mm, 16.0 mm–13.2 mm, and 13.2 mm–9.5 mm. The gradation distribution of the reduced-scale ballast particles is detailed in Table 1, and the sieving results are shown in Figure 1. Since the cleanliness of the ballast particle surface significantly affects the adhesion between polyurethane and the ballast [27], after screening, the ballast particles were placed in a water tank and rinsed with running water until no turbidity was observed. The washed ballast particles were then left to air dry outside the laboratory, and specimens were prepared only after their surfaces had completely dried.

#### 2.1.2. Polyurethane Materials

This experiment utilized isocyanate, polyol, and catalyst raw materials supplied by Shanghai Huafon Material Technology Institute (Shanghai, China). The mass ratio of isocyanate to polyol was 54:100, and the catalyst accounted for approximately 0.5% of the polyol’s mass. After thorough reaction, the raw materials formed an elastic cured body with uniform, fine cells and no structural defects such as cracks or voids, exhibiting excellent elastic deformation capacity and elongation at break.

Research by Zheng et al. [28] indicates that the mechanical properties of cured polyurethane materials, such as tensile, compressive, and adhesive strengths, are highly correlated with their foam density: the higher the density, the higher the tensile and compressive strengths of the cured polyurethane material. In this experiment, a polyol with a free-foam density of 180 kg/m^3^ was used to produce the cured polyurethane material.

#### 2.1.3. Geogrid

**Figure 2:**
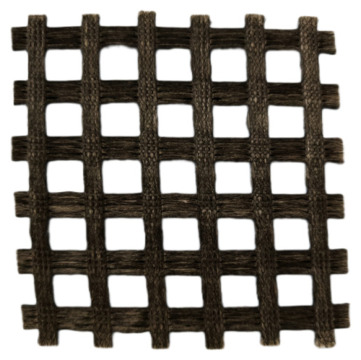
Biaxial warp-knitted polyester geogrid.

In this experiment, a biaxial warp-knitted polyester geogrid was selected as the reinforcement material for the PSB specimens, as shown in Figure 2. The geogrid is manufactured from high-strength polyester fiber raw materials using warp-knitted orientation technology, and is reinforced by wrapping and binding high-strength filaments at the intersecting nodes to create a robust, integrated geogrid structure. This geogrid not only possesses high tensile strength and low elongation but also exhibits excellent corrosion and aging resistance, enabling a strong interlocking effect with soil and rock materials. The specific physical parameters of the geogrid are as follows: strip width 10 mm, spacing 25 mm, mesh size 15 mm × 15 mm, ultimate tensile strength 30 kN/m, and elongation at break ≤ 10%.

#### 2.1.4. Specimen Preparation Procedure

Polyurethane materials generate significant expansion forces during the foaming process. Research by Qie et al. [29] indicates that when pouring PSTB, the expansion forces generated by the polyurethane can reach up to 40 kN. To prevent mold deformation caused by the expansion force generated during the polyurethane foaming process, this study employed high-strength cast iron test molds with an internal side length of 150 mm and a wall thickness of 5 mm. This ensures that the mold’s geometric dimensions remain constant throughout the specimen preparation process, thereby guaranteeing the consistency of the specimens’ final geometric shapes. The mold’s side plates, bottom plate, and top plate are all designed to be removable, with each plate bolted together to facilitate demolding after the specimen has been formed.

In addition, polyurethane materials exhibit high viscosity and significant exothermic heat release during the foaming and expansion process. To address the potential issue of specimens adhering to the mold caused by these factors, this study employed a method in which the inner surface of the mold was coated with a lubricant and then covered with an ultra-thin PE plastic film (0.01 mm thick) to produce the specimens. This method ensures the integrity and surface quality of the specimens upon demolding while preventing lubricant from penetrating into the specimens and causing adverse effects.

The amount of ballast used in a single standard PSB specimen is 5.17 kg, and the amount of polyurethane curing material is 450 g. When preparing geogrid-reinforced PSB specimens, the volume of the geogrid was accounted for by reducing the ballast content per specimen to 5.00 kg, while the amount of polyurethane curing material remained unchanged. The ballast content for each particle size range in a single specimen is shown in Table 2.

The procedure for preparing specimens is as follows:

(1) Preparation. Apply a uniform coat of lubricant to the inner walls of the mold and cover them with an ultra-thin PE plastic film, then assemble the mold. Weigh the ballast of four different particle size ranges according to Table 2, mix thoroughly, and load into the mold. Place the mold filled with ballast on a vibrating table and vibrate for 30 s to compact the ballast particles. Weigh the isocyanate and polyol raw materials separately; the mass ratio of the two is 54:100, with a total mass of 450 g.
(2) Casting the polyurethane. Cast the two polyurethane components and the catalyst into the hopper of the high-pressure grouting machine. Mix with a handheld high-speed mixer for at least 5 s. Once the two components are thoroughly mixed, immediately start the grouting machine to inject the slurry evenly into the mold from the top, ensuring sufficient bonding between the particles.
(3) Post-casting procedures. After casting is complete, cover the mold with a lid and secure it with bolts to prevent the polyurethane from expanding and overflowing during foaming. It is important to note that the high-pressure injection hose must be removed from the mold immediately after pouring. Then, quickly pour a large amount of injection machine cleaner into the hopper to flush the machine and hoses, preventing the foamed polyurethane from adhering to the interior and causing blockages. To maintain the polyurethane foaming temperature within the optimal range of 30–50 °C, place electric heaters on both sides of the mold and use an infrared thermometer to monitor the mold temperature at regular intervals.
(4) Demolding and curing. Demold the specimens 2 days after pouring, then place the specimens at the testing site in a room temperature of approximately 20 °C for curing for 7 days. No special curing conditions are required during this period.

**Figure 3:**
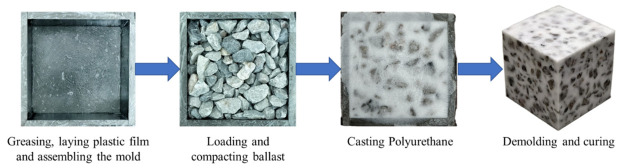
Procedure for preparing standard PSB specimens.

**Figure 4:**
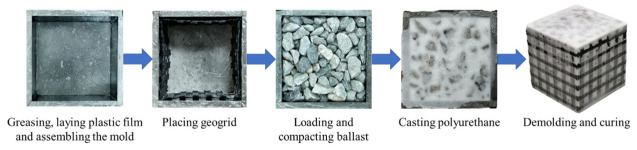
Procedure for preparing geogrid-reinforced PSB specimens.

**Figure 5:**
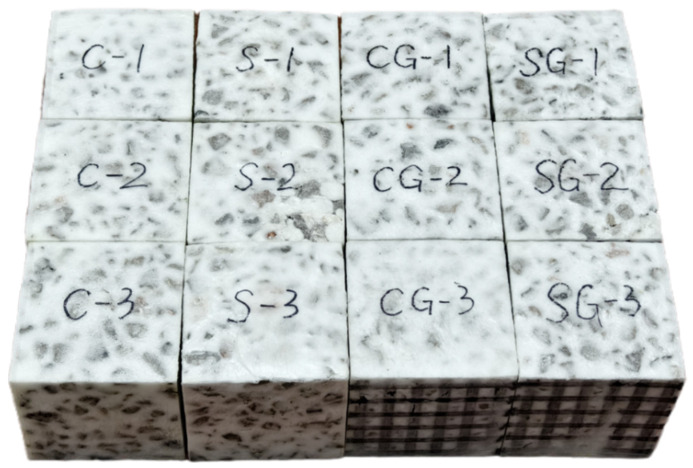
Finished PSB specimens (Left: standard specimens; Right: geogrid-reinforced specimens).

The process for preparing standard PSB specimens is shown in Figure 3. The process for preparing geogrid-reinforced specimens is essentially the same; the only difference is that, before filling the mold with ballast, the cut geogrid must be placed against the inner wall of the assembled mold. This process is shown in Figure 4. The finished specimens are shown in Figure 5.

### 2.2. Uniaxial Compression Test

The compressive properties of specimens can be evaluated through uniaxial compression testing. When conducting loading tests on viscoelastic materials such as polyurethane, the test results vary depending on the loading rate [30]. In this study, the loading rate was set to 2 mm/min. At this rate, the stress state of the specimens can be considered quasi-static, thereby preventing plastic deformation from affecting the test results.

**Figure 6:**
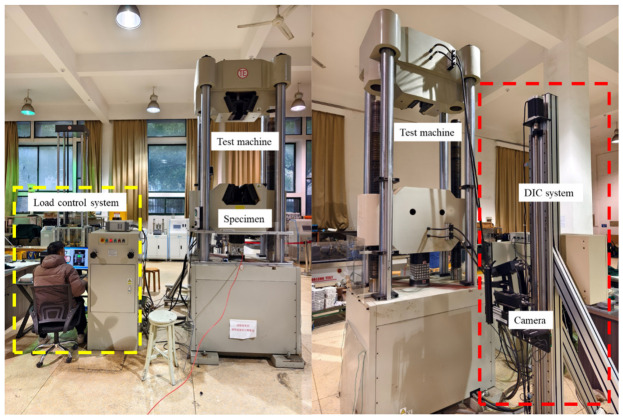
Test loading device.

From the same batch of cast specimens, three standard PSB specimens were labeled C-1, C-2, and C-3, respectively, while three geogrid-reinforced specimens were labeled CG-1, CG-2, and CG-3, respectively. The loading apparatus used was the high-stiffness tensile-compressive full-field strain testing system from Central South University (Model: Lisu/WAW-2000EDC), as shown in Figure 6. In addition, Digital Image Correlation (DIC) was used to record the deformation process of the specimens, and a high-precision displacement sensor was used to measure the lateral displacement changes of the specimens during compression to obtain lateral strain data and thereby calculate the Poisson’s ratio.

**Figure 7:**
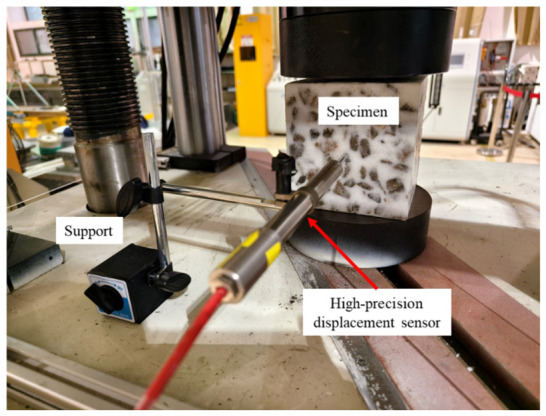
Uniaxial compression test site.

Loading was performed using a displacement control mode at a constant rate of 2 mm/min, and data on the vertical load and vertical displacement of the specimens were recorded during the loading process. After the test was completed, the specimens were removed from the loading machine, and the failure characteristics of the two groups of specimens were observed. The uniaxial compression test setup is shown in Figure 7.

### 2.3. Oblique Shear Test

**Figure 8:**
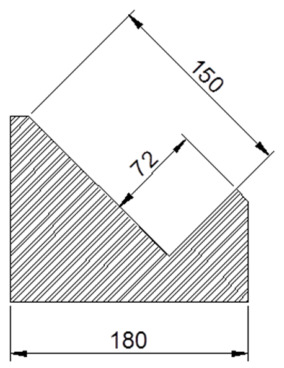
Dimensions of the fixture cross-section (unit: mm).

**Figure 9:**
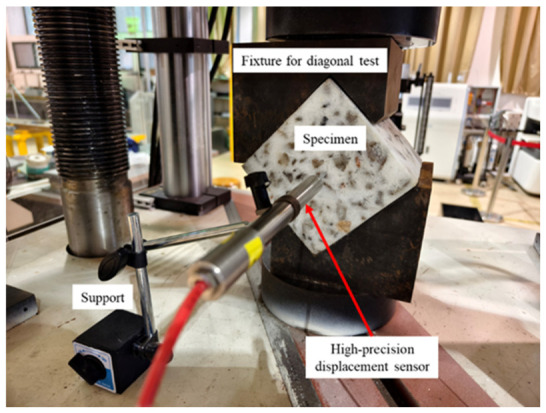
Oblique shear test site.

To obtain the shear mechanical properties of the specimens, this study conducted oblique shear tests. For testing the shear performance of 150 mm cubic specimens, a specialized 45° oblique shear cast iron fixture was designed, as shown in Figure 8. This apparatus consists of a pair of structurally identical fixtures arranged on the upper and lower sides, perpendicular to the cross-section shown in the figure, with a thickness of 150 mm. To investigate differences in shear performance across different shear planes, specimens from the same casting batch were selected. The standard PSB specimen was designated as S-1, while the geogrid-reinforced PSB specimen with the top surface as the shear plane (i.e., the side without geogrid reinforcement) was designated as SG-2. and the specimen with the shear plane on the side (i.e., the geogrid-reinforced surface) was labeled SG-3. The test employed a displacement-controlled loading method with a constant loading rate of 2 mm/min. Vertical load and displacement data for the specimens were automatically recorded by the loading machine, while lateral displacement data were obtained using a high-precision displacement sensor (Changsha Jinma Measurement and Control Technology Co., Ltd., Changsha, China). After the test, the specimens were retrieved, and the failure characteristics of the three types of specimens were observed. The on-site setup for the oblique shear test is shown in Figure 9.

## 3. Test Results

### 3.1. Uniaxial Compression Test Results

The formula for the unconfined compressive strength of a cube $f_{c}$ is:

(1) $$f_{c}=\frac{F_{c}}{A_{c}}$$

where $f_{c}$ is the unconfined compressive strength of the specimen (kPa) $F_{c}$ is the failure load of the specimen (kPa); $A_{c}$ is the compressed area of the specimen, and it is set to 22,500 mm^2^.

Due to the viscoelastic properties of polyurethane, no distinct failure stage was observed during loading; therefore, the failure load $F_{c}$ was taken as the peak load. The compressive strengths of the specimens in the uniaxial compression test are shown in Table 3. As can be seen from the table, the average compressive strength of the standard PSB specimens was 1145.8 kPa. However, after reinforcement with geogrids, the average compressive strength of the specimens increased to 1628.0 kPa, representing a 42.1% improvement compared to the specimens without geogrid reinforcement. This indicates that geogrids are highly effective in enhancing the compressive strength of the specimens.

**Figure 10:**
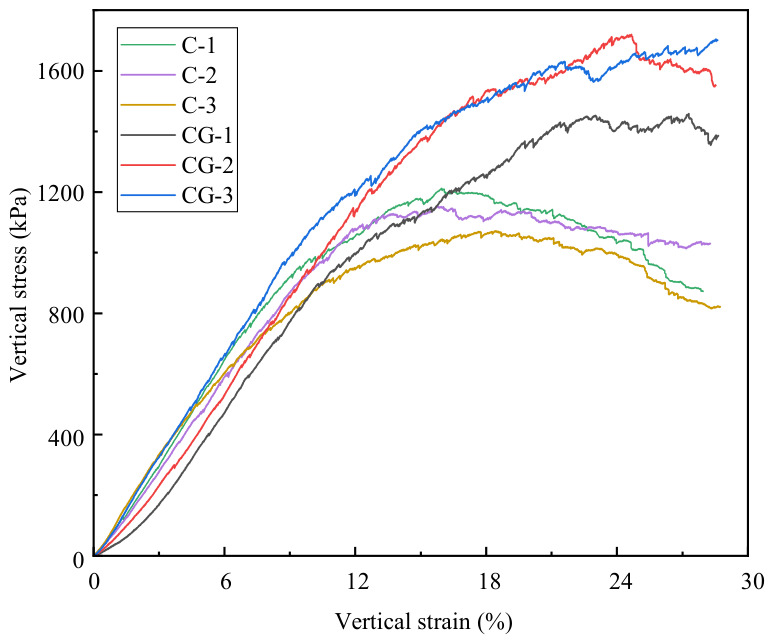
The stress–strain curves of the standard (C) and geogrid-reinforced (CG) specimens during uniaxial compression test.

Figure 10 shows the vertical stress–strain curves of two sets of PSB specimens during uniaxial compression testing. Before the vertical strain reached approximately 6%, both sets of specimens exhibited essentially identical stress–strain behavior, indicating that they were both in the linear elastic stage. Subsequently, the stress in both sets of specimens continued to rise with increasing strain, but the rate of increase gradually slowed down, and the stiffness of the specimens decreased to some extent. The decrease was more pronounced in the ordinary specimens compared to the geogrid-reinforced specimens. The peak load was reached when the vertical strain reached approximately 18%, after which the stress began to decline significantly. In contrast, the geogrid-reinforced specimens did not approach their peak load until the vertical strain reached approximately 24%, after which the stress fluctuated up and down with increasing strain. When the vertical strain reached approximately 30%, both sets of specimens were considered to have reached their ultimate bearing capacity, and the geogrid-reinforced specimens demonstrated higher strength compared to the standard specimens.

The formulas for the elasticity modulus E and Poisson’s ratio ν are:

(2) $$E=\frac{{∆\sigma }_{y}}{{∆\varepsilon }_{y}}$$

(3) $$\nu =\frac{{∆\varepsilon }_{x}}{{∆\varepsilon }_{y}}$$

where E is the elasticity modulus (MPa); ${∆\sigma }_{y}$ is the increase in vertical stress when vertical strain increases from 2.5% to 5% (MPa); the corresponding increment in vertical strain ${∆\varepsilon }_{y}=2.5\%$ (dimensionless); ν is Poisson’s ratio (dimensionless); ${∆\varepsilon }_{x}$ is the increase in transverse strain when vertical strain increases from 2.5% to 5% (dimensionless). The elastic moduli and Poisson’s ratios for the two sets of specimens were calculated as shown in Table 4.

Analysis of the data in the table shows that the average elastic modulus of the standard PSB specimens was 10.34 MPa, while that of the geogrid-reinforced specimens was 10.27 MPa; the elastic moduli of the two types of specimens were nearly identical. Similarly, there was no significant difference in the Poisson’s ratio between the two types of specimens, with an average value of approximately 0.27. This indicates that the geogrid does not provide a significant confining reinforcement effect on the specimens during the linear elastic stage. This may be because the geogrid does not come into direct contact with the ballast within the specimens; instead, it relies primarily on the bonding effect of the polyurethane. During the linear elastic stage, the deformation of the specimens is minimal and does not reach the tensile strength limit of the polyurethane. Therefore, under axial loading, the geogrid expands and deforms along with the specimen as a whole, and thus cannot exert a reinforcing effect at this stage.

Table 5 shows the maximum lateral displacement of the specimens under uniaxial compressive loading. As can be seen, the average maximum lateral displacement of the standard specimens was 26.53 mm, while that of the geogrid-reinforced specimens was 22.76 mm—a reduction of 14.2%. This indicates that the geogrid effectively restrains the lateral deformation of the specimens.

**Figure 11:**
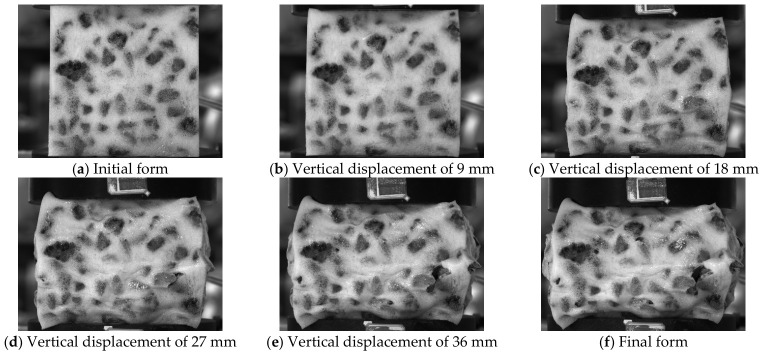
Deformation process of standard PSB specimens in uniaxial compression test.

Using DIC technology, continuous images were captured at fixed intervals to record the deformation of PSB specimens during uniaxial compression tests as loading progressed. Figure 11 shows the deformation of standard PSB specimens under different vertical displacements. Taking the C-3 specimen as an example, during the initial loading stage, the specimen’s deformation was minimal, primarily resulting from the compression of the voids in the polyurethane-cured material and the resulting compaction of the PSB particles. As the load gradually increases, the portion of deformation contributed by the polyurethane gradually decreases, while the skeleton formed by the ballast particles begins to bear more of the vertical load; simultaneously, the polyurethane bonding also plays a certain role. When the vertical displacement increases to 18 mm, the ballast near the specimen surface tends to be squeezed outward, causing wrinkles and bulges to appear on the polyurethane surface. Under continuous loading, the ballast within the specimen gradually migrated toward the exterior of the specimen perpendicular to the loading direction. Once the bonding strength of the polyurethane and the interparticle friction could no longer effectively resist lateral deformation, fine cracks began to form at the bonding interface. As the vertical displacement continued to increase to 36 mm, the cracks inside the specimen began to propagate in all directions. At this point, multiple cracks appeared on the surface of the polyurethane, and as the cracks developed, some ballast particles were squeezed out of the specimen surface, marking the specimen’s entry into the failure stage. The loading process continued until the vertical displacement reached approximately 45 mm, at which point significant outward bulging of the specimen’s sides could be observed, with the bulging in the middle of the specimen being the most pronounced. The polyurethane surface exhibited multiple cracks, and the internal ballast particles were squeezed out through the cracks, indicating that the specimen had approached its load-bearing capacity limit.

**Figure 12:**
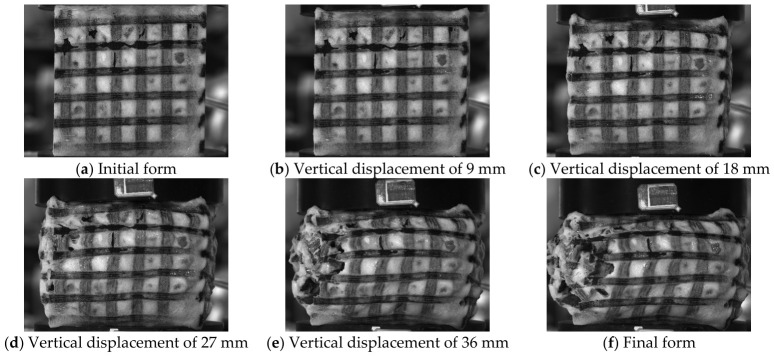
Deformation process of geogrid-reinforced PSB specimens in uniaxial compression test.

Figure 12 shows the deformation of geogrid-reinforced PSB specimens under uniaxial compression at different vertical displacements. Taking the CG-3 specimen as an example, the overall deformation of the specimen during the initial loading phase was essentially consistent with that of the ordinary specimen. This finding is corroborated by the similar patterns observed in the stress–strain curves of both specimens during the linear elastic stage. It can be observed that only a small amount of polyurethane exhibited slight bulging from the mesh openings of the geogrid. When the vertical displacement increased to 18 mm, the lateral expansion deformation of the specimen became progressively more pronounced. At this point, the geogrid gradually began to constrain the lateral deformation, while the polyurethane continued to be squeezed out and expanded through the geogrid’s apertures, causing the surface to become uneven. As the vertical load increased, the specimen exhibited significant lateral expansion, with ballast particles gradually migrating toward the outer surface, and ballast particles beneath the polyurethane layer protruding outward. Since the geogrid’s aperture size of 15 mm matches the average particle size of the ballast, it was able to limit the tendency of some ballast particles to bulge outward. When the vertical displacement reached 36 mm, the geogrid exhibited significant lateral bending deformation. Tears occurred at multiple polyurethane interfaces as they reached their tensile strength limit, and a small portion of the ballast was squeezed out through the geogrid apertures. The loading process continued until a vertical displacement of 45 mm was reached. At this point, it was observed that the overall lateral deformation of the geogrid-reinforced specimen was slightly smaller than that of the ordinary specimen, indicating that the geogrid played a certain role in constraining the specimen’s deformation.

**Figure 13:**
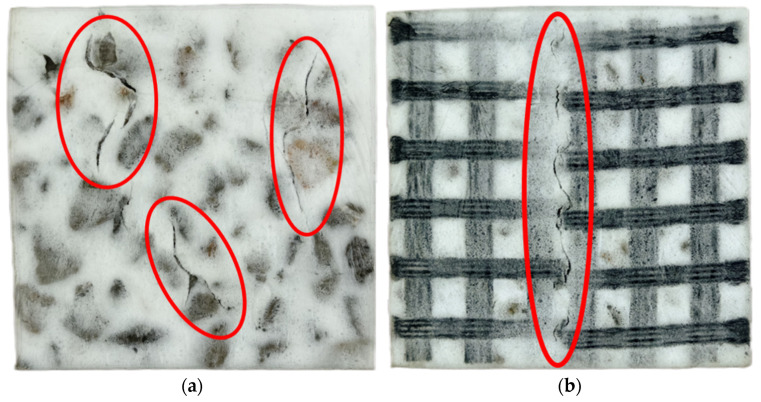
Crack patterns in specimens after uniaxial compression test: (**a**) standard specimen; (**b**) geogrid-reinforced specimen.

The crack patterns of the two sets of specimens after uniaxial compression testing are shown in Figure 13, where the red circles indicate the crack positions. Taking the ordinary PSB specimen C-3 as an example, the figure shows that under axial compressive loading, the surface ballast particles on the side of the ordinary specimen experienced dislocation and slippage. A distinct tear occurred at the bond interface between the ballast and the polyurethane, and the crack propagated along this interface. In contrast, the crack patterns of the geogrid-reinforced PSB specimens are relatively uniform. Taking specimen CG-3 as an example, since the center of the specimen experiences the greatest lateral deformation, a through crack appears only along the central axis, with no significant cracks observed elsewhere.

### 3.2. Oblique Shear Test Results

**Figure 14:**
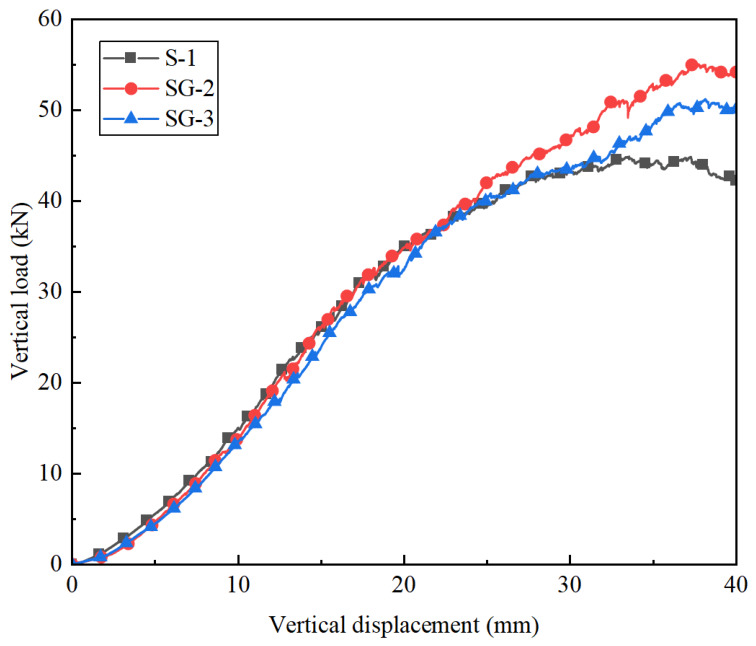
Vertical load–displacement curves during the oblique shear test.

The vertical load–displacement curves of the specimens in the oblique shear test are shown in Figure 14. Observation of the curves reveals that the deformation trends of the three specimens are generally consistent. Before the vertical displacement reached approximately 25 mm, the three curves were essentially superimposed, further confirming the characteristic that geogrids are unable to provide reinforcement during the linear elastic stage of the specimens. Subsequently, as loading continued, the vertical loads on the three specimens gradually diverged. When the vertical displacement reached approximately 36 mm, the loads on each specimen approached their peak values, after which they fluctuated up and down as displacement increased. The loading process continued until the vertical displacement reached approximately 40 mm, at which point the specimens were considered to have failed. Compared to the standard PSB specimen, the two geogrid-reinforced specimens exhibited higher peak loads. Specimen SG-2, with the shear plane on the top surface, had the highest peak load of 55.19 kN; specimen SG-3, with the shear plane on the side, had the second-highest at 51.21 kN; while the standard PSB specimen S-1 had the lowest, at 44.86 kN. When the shear plane is the top surface, the longitudinal strips of the geogrid are subjected to tensile stress due to the lateral expansion of the specimen, while the transverse strips are primarily subjected to shear stress; when the shear plane is the side surface, the longitudinal strips of the geogrid are almost stress-free, while the transverse strips are subjected to the combined effects of shear and bending in the direction perpendicular to the shear plane. Since geogrids primarily resist tensile loads rather than bending or shear forces, the specimen exhibits higher overall shear strength when the shear plane is the top surface.

**Figure 15:**
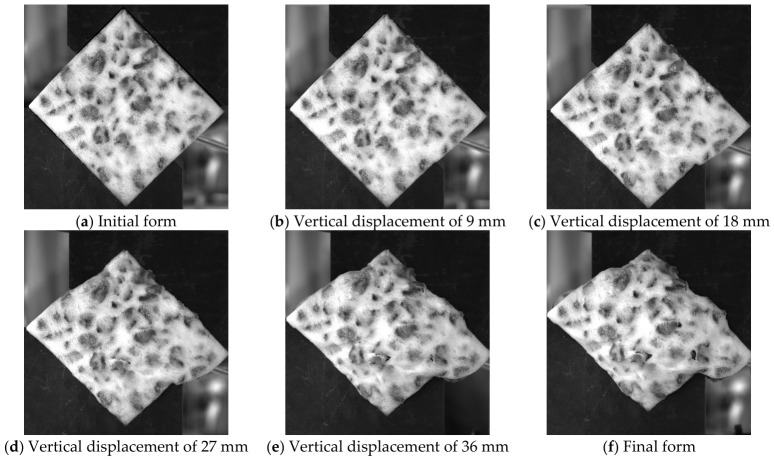
Deformation process of the S-1 specimen in the oblique shear test.

**Figure 16:**
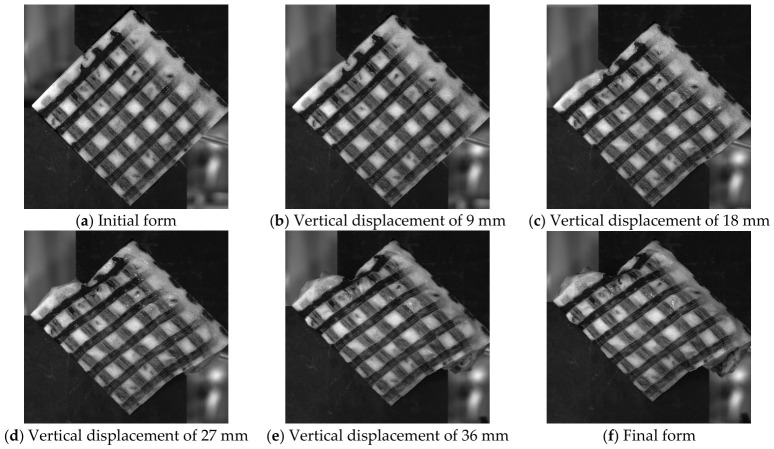
Deformation process of the SG-2 specimen in the oblique shear test.

**Figure 17:**
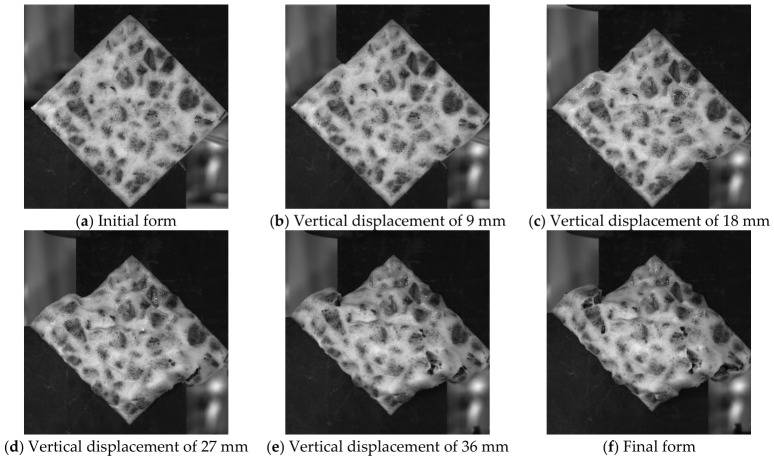
Deformation process of the SG-3 specimen in the oblique shear test.

Using DIC technology, the deformation of the three PSB specimens during the oblique shear test was continuously recorded at fixed intervals as vertical displacement increased. The deformation processes of S-1, SG-2, and SG-3 are shown in Figure 15, Figure 16 and Figure 17, respectively. As can be seen from the three sets of images, the three specimens exhibited similar deformation patterns under loading. When the vertical displacement reached 9 mm, the specimens exhibited only slight slippage along the oblique shear plane; it is believed that the specimens were still in the linear elastic stage at this point. As loading continued, the specimens began to exhibit significant slippage along the shear plane; however, no significant cracking was observed until the vertical displacement reached 27 mm, indicating that the polyurethane continued to consolidate the ballast particles and constrain the overall deformation of the specimens. When the vertical displacement reached 27 mm, distinct cracks began to form in certain areas near the shear plane on the non-geogrid-reinforced surfaces of the S-1 and SG-3 specimens. The ballast particles detached from the polyurethane and began migrating outward from the specimen, resulting in significant lateral expansion; the lateral expansion of the geogrid-reinforced specimens was slightly better than that of the standard specimens. When the vertical displacement reached 36 mm, significant extrusion of ballast particles occurred on the non-geogrid-reinforced surfaces of both the S-1 and SG-3 specimens, and the previously formed cracks further expanded and extended. In contrast, the geogrid-reinforced surface of the SG-2 specimen remained crack-free due to the confining effect of the geogrid. After loading was completed, the failure patterns of the three specimens were essentially consistent: significant displacement occurred along the shear plane, and all three specimens exhibited noticeable lateral expansion perpendicular to the shear plane. For the standard specimen, the polyurethane near the shear plane suffered significant tearing failure, with ballast particles being squeezed out through the cracks; In contrast, for the two geogrid-reinforced specimens, the polyurethane on the reinforced surface did not tear. The ballast particles beneath the polyurethane layer were not forced out of the specimen surface due to the confinement provided by the geogrid. This indicates that under shear loading, the geogrid also served to constrain the lateral deformation of the specimen and enhance its overall load-bearing capacity.

**Figure 18:**
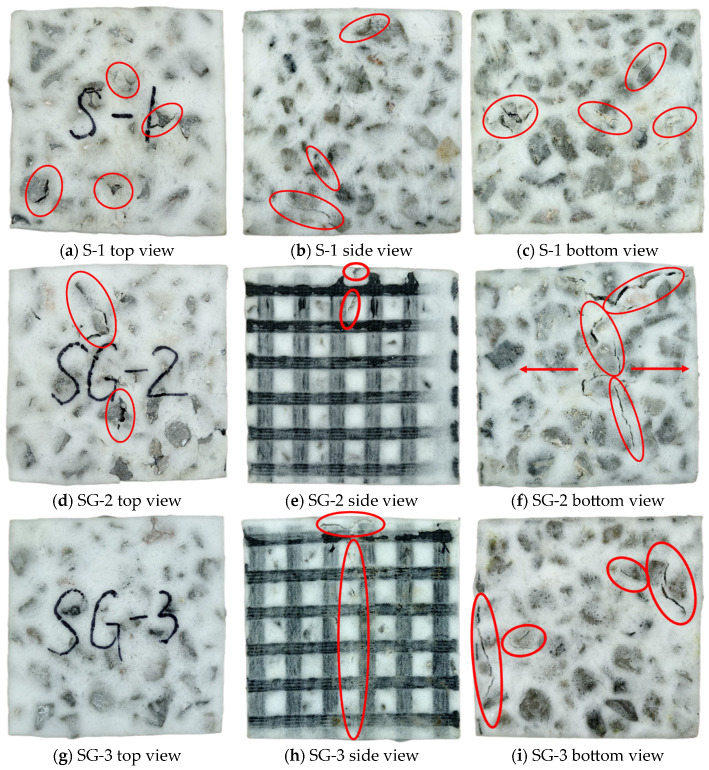
Crack morphology of specimens after the oblique shear test.

Figure 18 shows the crack patterns of the three specimens after the oblique shear test, where the red circles indicate the crack positions. As can be seen from the figure, all three specimens exhibit cracks of varying lengths near the shear plane, with a higher concentration of cracks on the top and bottom surfaces. The cracks all propagate along the interface between the ballast particles and the polyurethane. For the two geogrid-reinforced specimens, when the shear plane was the top surface, only a small crack appeared at the centerline near the top surface of the geogrid-reinforced face; when the shear plane was the side surface, a through-crack was observed along the centerline of the geogrid-reinforced face in the direction of shear.

## 4. Finite Element Simulation of the PSB

### 4.1. Geometric Modeling

#### 4.1.1. Three-Dimensional Random Aggregate Mesoscopic Model

When conducting finite element analysis of large-scale models, researchers often treat PSB as an isotropic homogeneous body to save time and improve computational efficiency. However, such assumptions overlook the mesoscale interactions between materials. Nevertheless, due to the computational characteristics of the finite element method, there has been little research on using finite element simulation to study the mesoscale behavior of PSB. In this section, drawing on the modeling concepts for microstructural aggregates in concrete, we establish separate models for the polyurethane matrix and three-dimensional random ballast aggregates, assign distinct material properties to each, and define the interactions between them. This results in a two-phase medium model composed of the polyurethane matrix and ballast particles, with the aim of obtaining simulation results that are more accurate than those from homogeneous solid models.

Among existing simulation methods, aggregates can be classified into spherical, ellipsoidal, and random polygonal aggregates based on their shape. Early simulations of aggregate particles using the finite element method employed spherical particles [31], which are simple in shape and offer high computational efficiency. However, the smooth and flat surface of spherical particles fails to capture the characteristics of irregular aggregate surfaces, resulting in poor adhesion to the matrix. Compared to spherical aggregates, ellipsoidal aggregates have a geometric shape that more closely resembles real-world conditions. However, due to their relatively smooth surfaces, simulations of the contact state between ellipsoidal aggregates and the matrix are not entirely accurate. Polygonal aggregates most closely resemble the actual shapes of aggregates and yield more precise simulation results. Zhao et al. [32] employed a random convex polyhedron model to simulate the irregular features of aggregate surfaces. This approach more closely approximates the geometric shape and spatial distribution of aggregates under actual conditions, offering higher computational accuracy and superior visualization. However, due to the highly irregular shapes of random convex polyhedrons, computational efficiency is low, and the model may suffer from poor convergence.

Because ballast particles have sharp edges, varied shapes, and significant anisotropy, their irregular morphology becomes a key factor affecting computational accuracy. On the one hand, this irregularity leads to uneven force transmission and distribution [33]; on the other hand, it constrains particle displacement and rotation through interlocking edges, thereby imparting strength and elasticity to the ballast bed [34]. Therefore, accurately modeling the geometry of ballast particles is crucial for ensuring the accuracy of numerical calculations [35].

**Figure 19:**
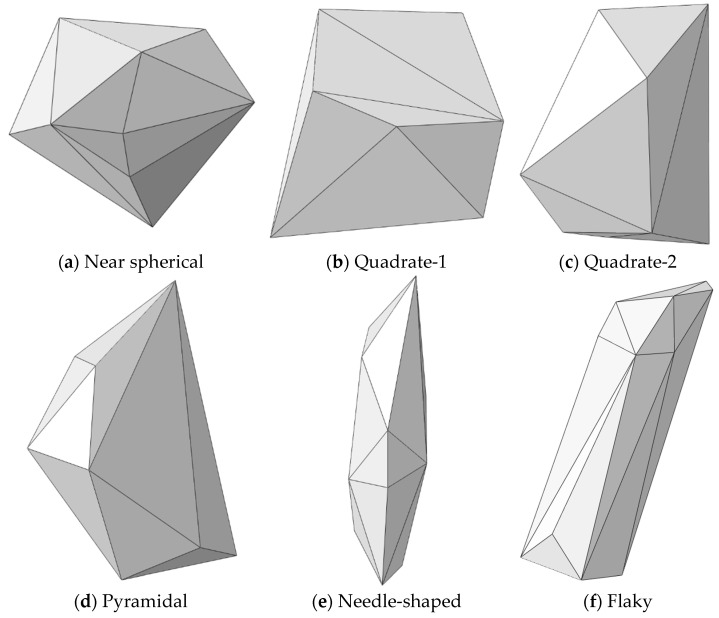
Ballast particle templates used in FEM.

In this study, the three-dimensional geometric features of real crushed stone were scanned and simplified to generate six convex polyhedral templates for ballast particles with varying roundness and aspect ratios, as shown in Figure 19. The geometric parameters for each particle are listed in Table 6.

To reproduce the discrete characteristics of the particle gradation in actual PSB specimens, this study requires the generation of a large number of ballast particles of varying sizes and randomly distributed positions within the computational model, based on the aforementioned predefined ballast shape templates, in order to construct a three-dimensional microstructure model consistent with real-world conditions. However, the finite element analysis software ABAQUS 2022 cannot generate components with random sizes and positions on its own. Therefore, this study employs Python 3.13 programming to perform secondary development on the Abaqus software, thereby enabling the creation of a PSB specimen model with three-dimensional random aggregates that account for gradation and real-world geometric characteristics. The specific steps for generating the random aggregate model are as follows:

(1) Definition of Basic Model Parameters

Define basic parameters such as the geometric dimensions of the specimens, the total target aggregate mass, the material densities of the track ballast and polyurethane, the aggregate size ranges, and the corresponding mass distributions of aggregates across different size ranges. These parameters provide a computational basis for estimating the quantity of aggregates to be generated, ensuring that the final aggregate gradation matches the gradation of track ballast particles in the actual PSB specimens.

(2) Estimation of Aggregate Quantities for Each Size Range

Based on the parameters defined in Step (1), first calculate the total aggregate mass for each size range using the total aggregate mass and the mass percentage of aggregates in each size range. then take the average of the upper and lower limits of the corresponding size range as the average particle size for that range. Treating the aggregate as approximately spherical particles, the volume of a single equivalent spherical aggregate is estimated using the average particle size. Combining this with the density of the ballast material yields the estimated mass of a single aggregate particle, and finally, the estimated quantity of aggregate to be generated for that size range is obtained.

(3) Generation of Random Aggregate Characteristics

Since larger-sized aggregates have a greater impact on spatial occupancy, prioritizing their placement can prevent the issue where small aggregates are generated first, making it impossible to place larger ones. Following the order of aggregate size distribution from largest to smallest, a random function is used to randomly select one of six pre-imported track ballast particle shape templates with different roundness and aspect ratios, simultaneously generating a random aggregate particle size within the current size range. A scaling ratio is calculated based on the target particle size and the template’s original maximum geometric dimensions. Finally, within the specimen’s spatial boundaries, the center coordinates and spatial rotation angle of the aggregate are randomly generated to determine the parameters of the virtual minimum circumscribed sphere corresponding to the aggregate particle.

(4) Aggregate Placement and Overlap Check

Based on the center coordinates and radius of the virtual minimum circumscribed sphere of the newly generated aggregate, a spatial position check is performed against the virtual circumscribed spheres of all previously placed aggregates: If the newly generated sphere does not overlap with any existing sphere, the placement is confirmed as valid; the aggregate information is stored in the results list, the placement is completed, and the process returns to step (3) to generate the next aggregate; If an overlap occurs, the current position is discarded, and new random coordinates and angles are generated to attempt placement. If no non-overlapping position can be found after the maximum number of attempts is reached, the generation of the current aggregate is abandoned; when all aggregates within the specified size range have been placed or the estimated generation quantity is reached, the aggregate generation process stops, and a parameter list of all successfully placed aggregates is output.

(5) Geometric Component Creation

Create a 2D sketch of the polyurethane matrix and use extrusion to generate a 3D polyurethane matrix component matching the specimen dimensions; subsequently, read the information for each successfully placed aggregate from the parameter list in sequence, and based on the selected ballast template, generate 3D aggregate components with corresponding shapes according to the calculated scaling ratio and rotation angle.

(6) Model Assembly and Boolean Operations

Import the generated polyurethane matrix part and all aggregate parts into the assembly module. Based on the center coordinates determined during the generation of each aggregate, translate the aggregates from the assembly’s default coordinate origin to their target positions, and perform spatial rotation positioning according to the random rotation angles obtained during generation. After positioning all aggregates, perform a Boolean union operation on all individual aggregate components to obtain a complete aggregate geometric model. Then, using the merged aggregate model as a cutting tool, perform a Boolean cut operation on the polyurethane matrix to ultimately obtain a microscopic numerical model of the PSB, which includes both the ballast aggregates and the polyurethane matrix.

**Figure 20:**
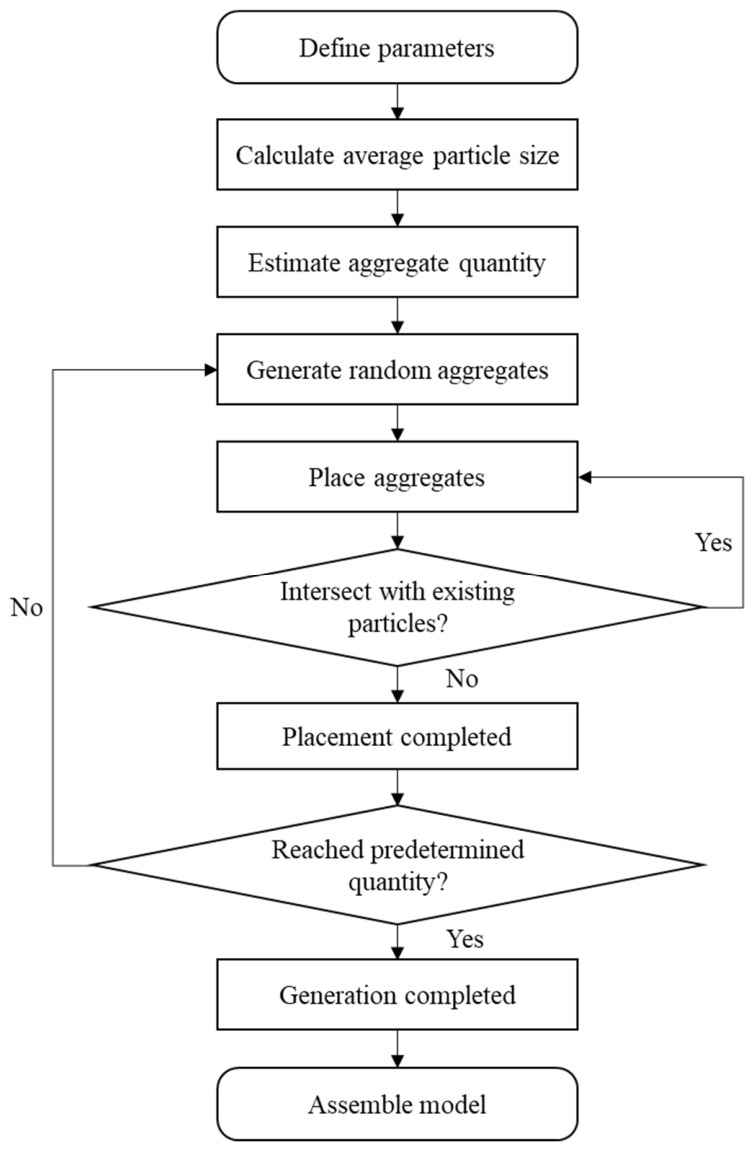
Flowchart of the algorithm for generating a random aggregate model.

**Figure 21:**
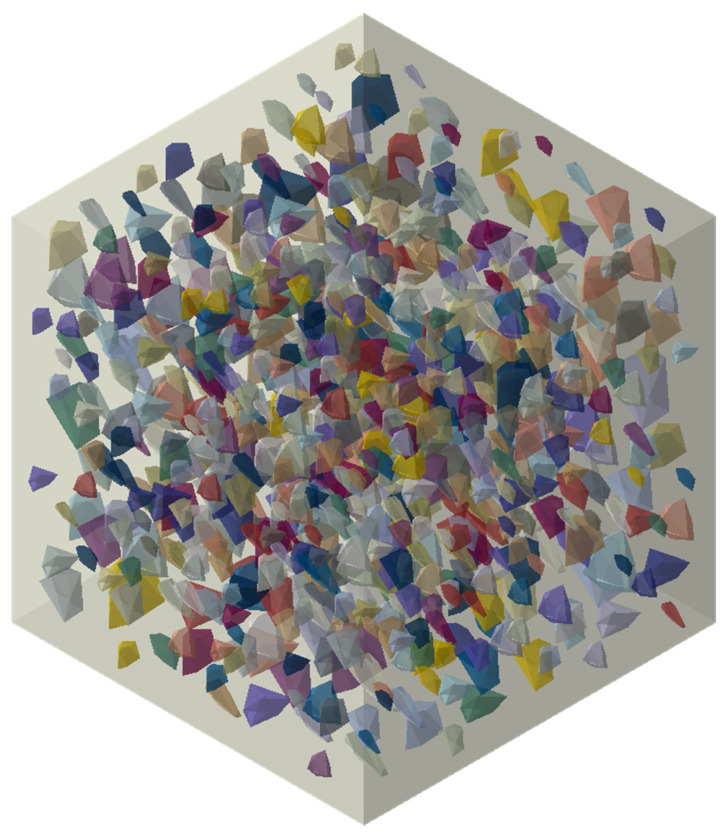
Rendering of a finite element model of a PSB specimen (perspective view).

Figure 20 illustrates the algorithmic workflow for generating a random aggregate model. A perspective view of the finite element model of the PSB specimen is shown in Figure 21.

#### 4.1.2. Geogrid Model

**Figure 22:**
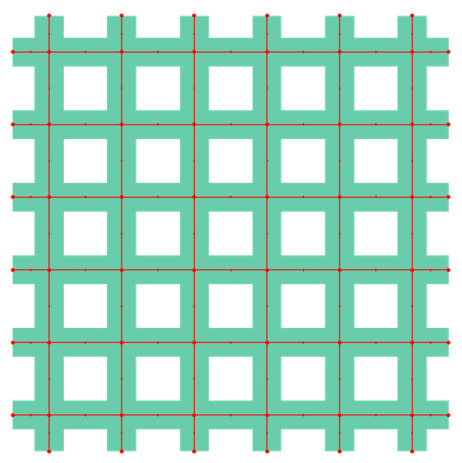
FEM model of geogrid.

**Figure 23:**
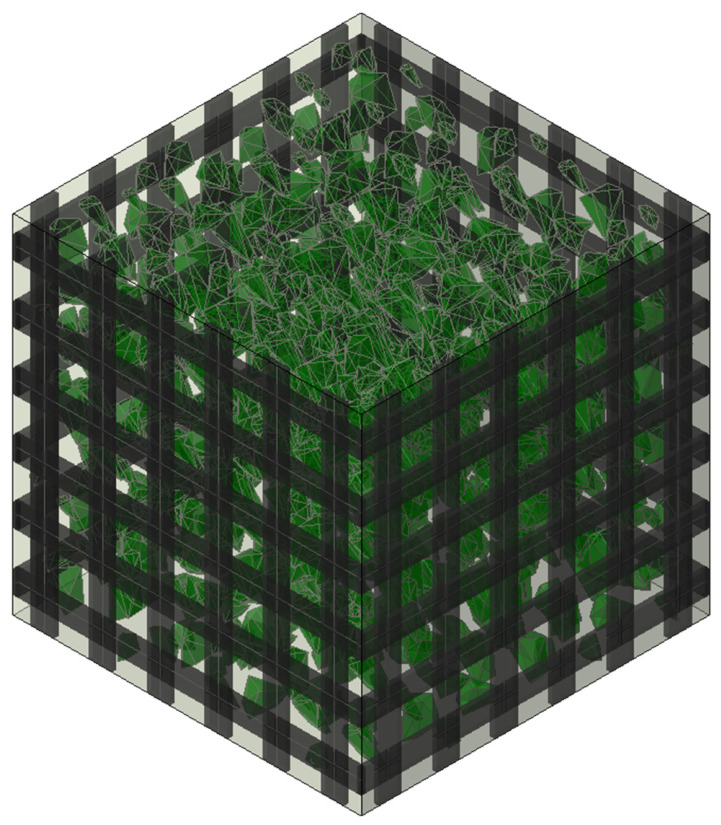
FEM model of geogrid-reinforced PSB specimen (perspective view).

Based on the previously established three-dimensional random aggregate microstructure model, a numerical model of geogrid-reinforced PSB was developed. For computational convenience, a two-node, six-degree-of-freedom beam element was used to simulate the geogrid. The center-to-center spacing of the geogrid was 25 mm, with a mesh size of 15 mm × 15 mm and a thickness of 2 mm. The finite element model of the geogrid is shown in Figure 22. In the figure, the red lines represent the beam elements actually involved in the calculation, while the green lines represent the cross-sections defined with material properties. A perspective view of the final finite element model of the geogrid-reinforced PSB specimen is shown in Figure 23.

### 4.2. Definition of Model Parameters

#### 4.2.1. Material Parameters

(1) Ballast Materials

Ballast particles are typically composed of materials such as basalt and granite. They are classified as quasi-brittle materials; when subjected to compression, they sequentially undergo a process of stiffness increase, stabilization, and eventual failure. As shown by the test results in Section 2, under uniaxial compression and oblique shear loads, the ballast particles themselves do not fracture; therefore, they can be simplified as ideal linearly elastic materials during the modeling process. In this model, the density of the ballast material is set to 2700 kg/m^3^, the Young’s modulus to 55 GPa, and the Poisson’s ratio to 0.2.

(2) Polyurethane Materials

Unlike traditional materials that follow Hooke’s law, polyurethane elastomers exhibit distinctly nonlinear mechanical properties. They can withstand reversible deformations far greater than those of conventional materials and display non-elastic characteristics such as the Mullins effect—where stress softening occurs after the initial loading—and hysteresis [36]. During large deformations, the material’s volume remains nearly constant, with a Poisson’s ratio close to 0.5, and its mechanical behavior is influenced by factors such as temperature and loading rate. Treating polyurethane elastomers as fully elastic hyperelastic materials is one of the most common approaches to modeling this class of materials, with the Mooney–Rivlin model being the most widely used among them [37].

In theory, to select an appropriate hyperelastic constitutive model, it is necessary to first obtain basic mechanical test data under six pure strain states: uniaxial tension and compression, isotropic biaxial tension and compression, and plane-strain tension and compression [38]. If the material is considered incompressible, the above six tests can typically be simplified to three types: uniaxial tension, isotropic biaxial tension, and plane strain tension, which are used to determine the material’s hyperelastic constitutive relationship [39].

Since the strain of the specimen will reach 30% during the entire uniaxial compression test—representing significant deformation—this study employs the Mooney–Rivlin constitutive model to assign hyperelastic material properties to the polyurethane material in order to accurately simulate its mechanical behavior. After repeated adjustments and trials, the coefficients $C_{10}$ and $C_{01}$ were finally set to 1.5 and 0.8 MPa, respectively, and the material compression coefficient $D_{1}$ was set to 0.35. The Maxps failure criterion, which is controlled by the maximum principal stress, was used to account for plasticity in the material. The maximum principal stress was set to 12 MPa, and a displacement-based damage evolution was defined, with the failure displacement set to 0.8 mm.

Regarding density, since the open-cell density of polyurethane is lower than the actual density achieved under the combined effects of ballast and mold pressure during foaming, the PSB specimens prepared in Section 2 were weighed. The average mass was approximately 5.67 kg. Using the total volume of the specimens, the mass of the PSB, and the density, calculations yielded a density of approximately 336 kg/m^3^ for the polyurethane-cured material.

(3) Geogrid Material

In simulation analyses, only the linear elastic properties of geogrids are typically considered. In this model, the geogrid was modeled using a tension-only elastic element characterized by its axial stiffness (EA). The moment of inertia of the geogrid material is 2 × 10^−9^ kg·m^2^, the Young’s modulus is set to 200 GPa, and the Poisson’s ratio is set to 0.25 [40]. Due to its low flexural rigidity, the element does not resist compression or bending.

#### 4.2.2. Other Parameters

In this paper, a contact model based on cohesive forces is adopted to account for the interaction between ballast aggregates and polyurethane. The normal stiffness $K_{nn}$, as well as the tangential stiffnesses $K_{ss}$ and $K_{tt}$, are all set to 1 × 10^8^ N/m^3^, and it is specified that cohesive forces apply only to nodes in the initial contact state. The outer surface of the ballast aggregate particles is designated as the primary surface, while the inner surface of the polyurethane matrix is set as the secondary surface to create a non-constrained contact and assign the aforementioned contact properties.

Due to the highly irregular shape of the aggregate component, C3D10M elements were used for meshing with a mesh size of 5 mm, and the distortion control option was enabled. For the polyurethane matrix, C3D8R hexahedral elements were used with a mesh size of 5 mm, and the element deletion option was enabled to remove failed elements and simulate the fracture of the polyurethane material.

### 4.3. Analysis of Simulation Results

#### 4.3.1. Displacement Analysis

**Figure 24:**
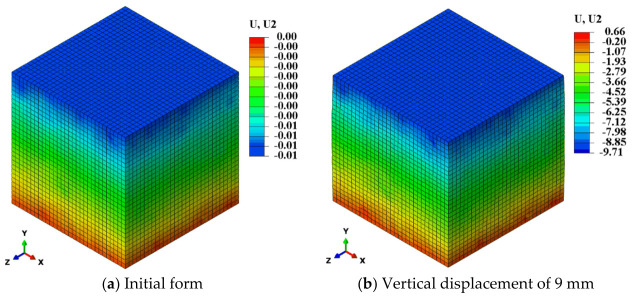

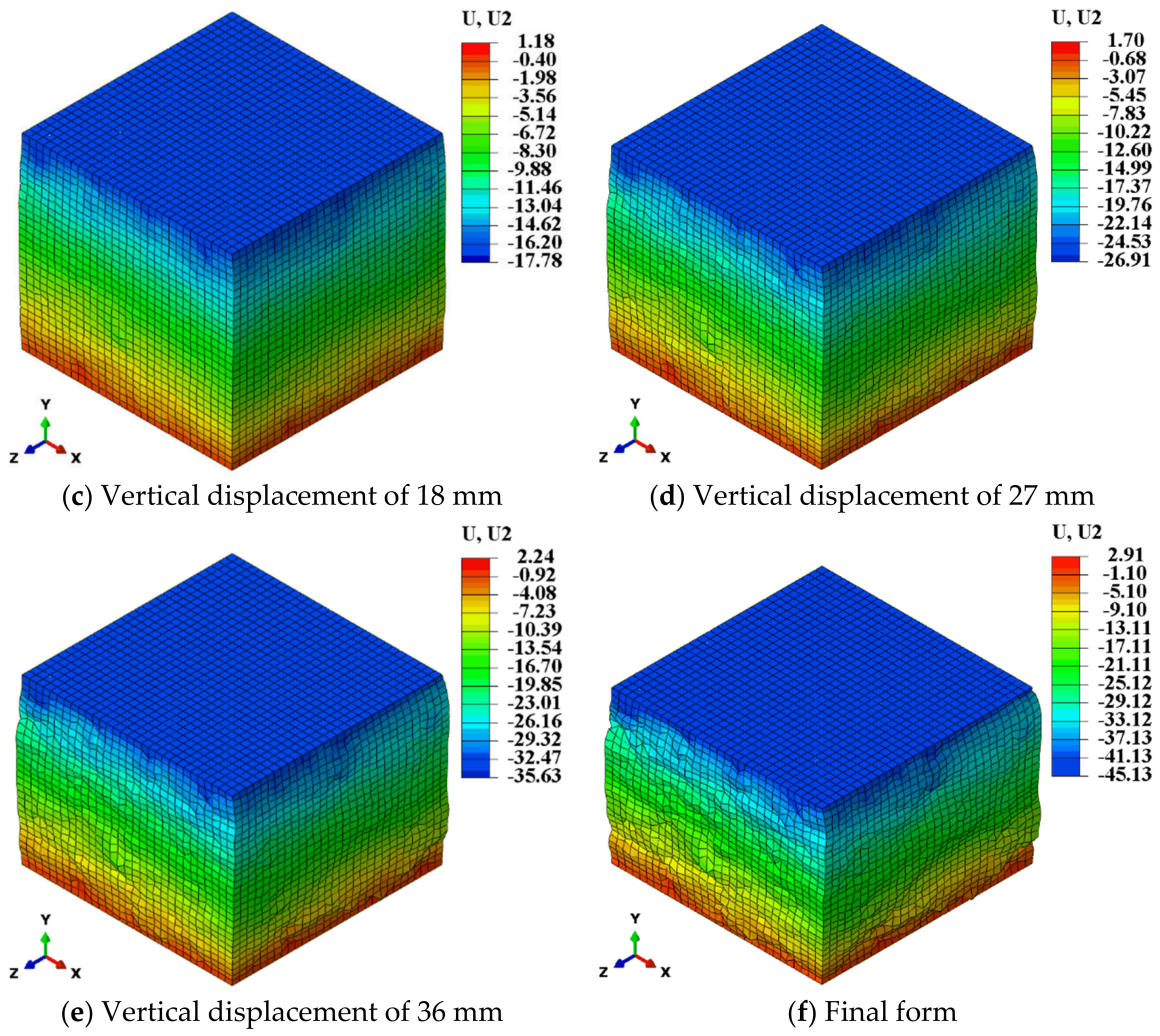
Contour plot of the overall deformation process of a standard specimen during a uniaxial compression simulation test (unit: mm).

Figure 24 shows the deformation process of a standard PSB specimen, modeled using a three-dimensional random aggregate model, during a simulated uniaxial compression test. As shown in the figure, the overall deformation of the standard PSB specimen under an axial unconfined load is generally consistent with the results of actual tests. As the vertical displacement increases, the specimen gradually undergoes lateral expansion. When the vertical displacement reaches approximately 27 mm, the ballast near the specimen surface tends to be squeezed outward, and distinct wrinkles and bulges gradually appear on the polyurethane surface. Upon reaching a vertical displacement of 45 mm at the end of loading, some ballast particles can be seen being squeezed out of the polyurethane specimen surface.

**Figure 25:**
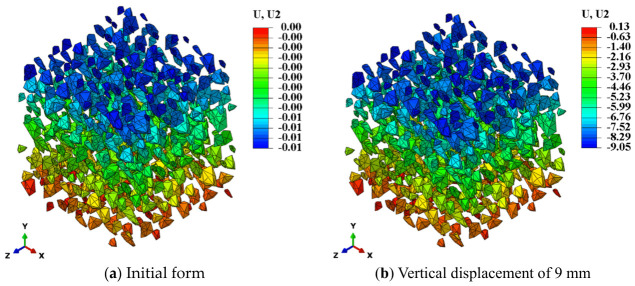

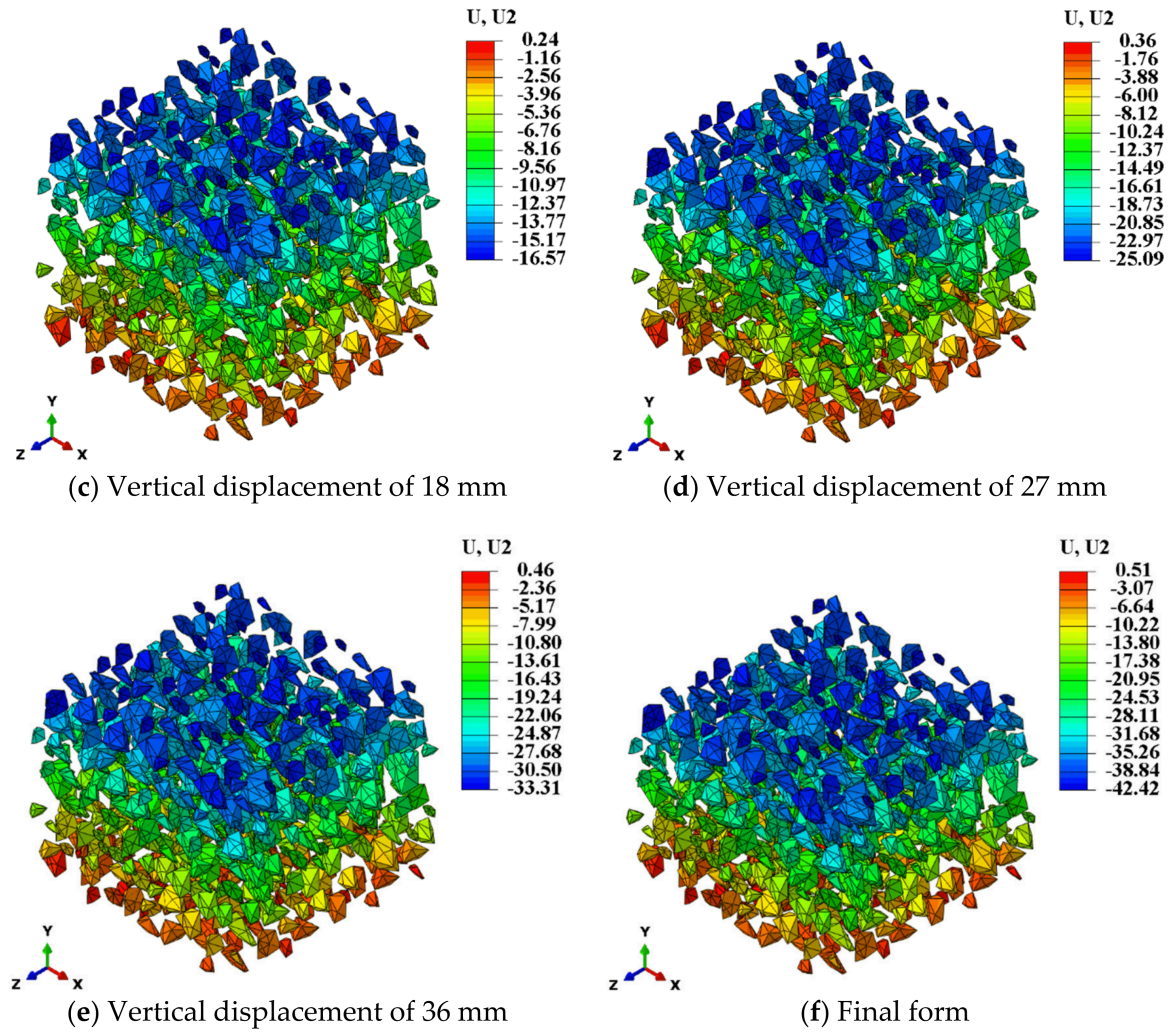
Contour plot of internal ballast particle displacement in standard specimens during uniaxial compression simulation test (unit: mm).

Figure 25 illustrates the displacement of the internal ballast particles. As vertical displacement increases, the ballast particles gradually become compacted, with a significant amount of ballast from the center of the specimens migrating toward the periphery. A comparison of the vertical displacements of the polyurethane matrix and the ballast particles at the same incremental step reveals that the vertical displacement of the ballast particles is slightly smaller than that of the polyurethane matrix, further corroborating the phenomenon of aggregate particle lateral migration.

**Figure 26:**
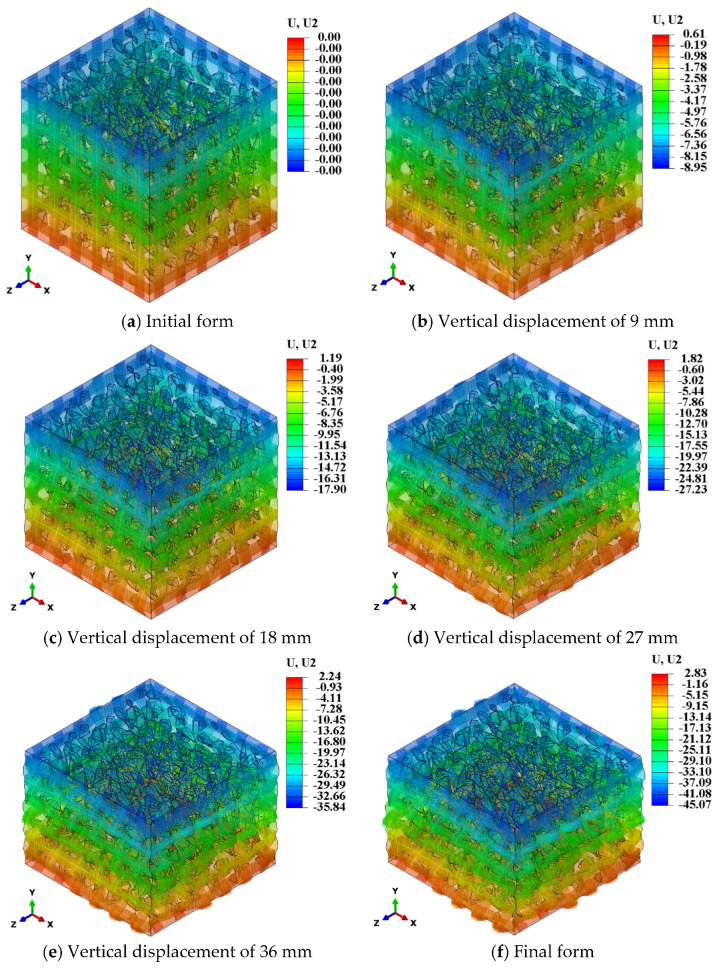
Contour plot of the overall deformation process of geogrid-reinforced specimen in uniaxial compression test (unit: mm).

Figure 26 illustrates the overall deformation process of the geogrid-reinforced specimen. It can be observed that the surface of the polyurethane matrix also exhibits uneven wrinkles due to the lateral migration of internal ballast particles toward the outer surface of the specimen, causing the geogrid components to bend outward and expand accordingly. It can be concluded that both the model of the PSB specimen established using a random aggregate model and the model of the geogrid-reinforced PSB specimen simulated using beam elements effectively reflect the actual deformation process of the specimens.

#### 4.3.2. Force Conditions Analysis

**Figure 27:**
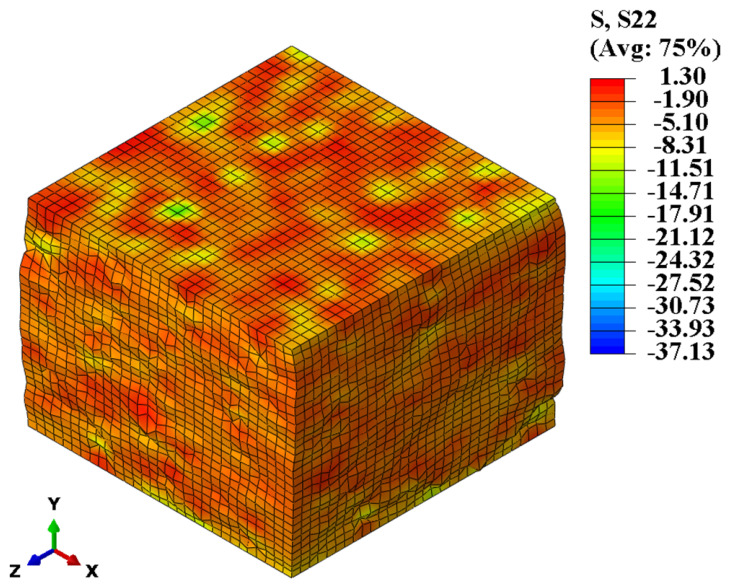
Contour plot of maximum stress after loading of standard specimens (unit: MPa).

Figure 27 shows the maximum stress contour plot of a standard specimen created using a three-dimensional random aggregate model during the loading process. It can be observed that the maximum stress on the top surface of the specimen is approximately 1.3 MPa (i.e., 1300 kPa), which is relatively close to the average maximum stress of 1145.8 kPa for specimens in uniaxial compression tests. The scattered light-colored patches visible in the figure are caused by stress concentrations resulting from the contact between the polyurethane and the ballast particles within the specimen.

**Figure 28:**
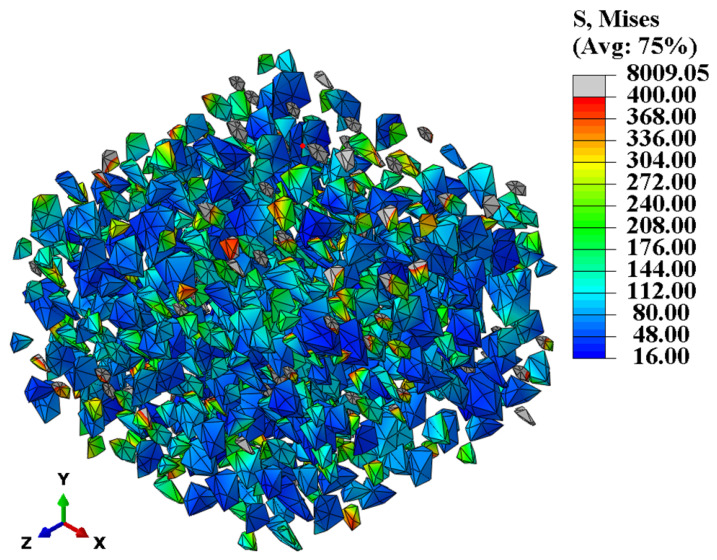
Stress contour plot of internal ballast particles in a standard specimen after loading (unit: kPa).

The stress contour plot of the ballast particles inside the specimen is shown in Figure 28. It can be observed that some ballast particles near the specimen boundary exhibit higher stress. This is likely because these particles have detached from the polyurethane bond and are about to be extruded from the specimen surface, thereby experiencing greater stress at the contact interface. The stress distribution of the ballast aggregate particles in the center of the specimen is relatively uniform, and the skeleton formed by these particles bears the primary external load.

**Figure 29:**
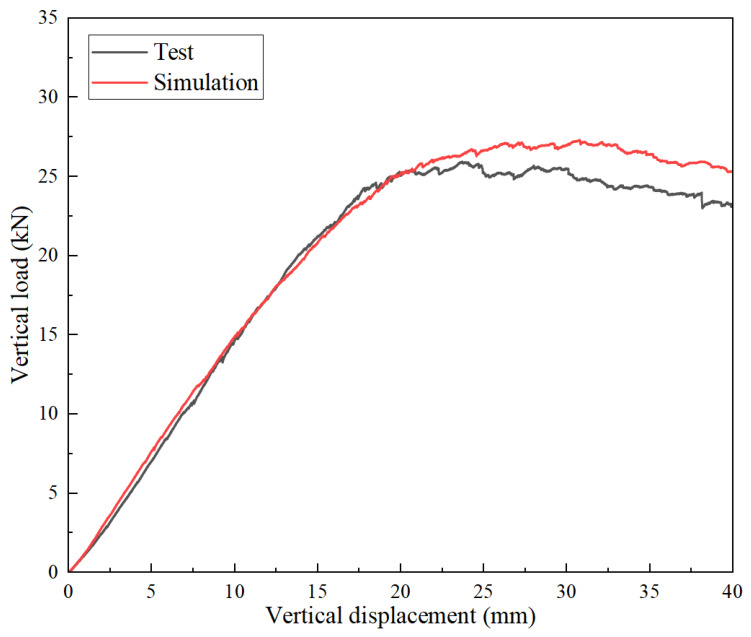
Comparison of test results with numerical simulation results.

A comparison of the results from uniaxial compression laboratory tests and numerical simulations is shown in Figure 29. As can be seen, the results of the uniaxial compression simulations using the random aggregate model show a high degree of agreement with the laboratory test results; therefore, the calibrated material parameters are considered reasonable.

## 5. Discussion

### 5.1. Effect of Geogrid Reinforcement on Compressive Behavior

The experimental results demonstrate that geogrid reinforcement increased the average compressive strength of the polyurethane-solidified ballast specimens from 1145.8 kPa to 1628.0 kPa, corresponding to an increase of 42.1%, while reducing the maximum lateral displacement by 14.2%. These findings are generally consistent with previous studies on geogrid-reinforced ballast, which reported that geogrids improve the load-bearing capacity and reduce permanent or lateral deformation by restraining particle movement and mobilizing particle–geogrid interlocking [21,22]. Prasad and Hussaini [23] also observed that the combined use of polyurethane and geogrid reduced the vertical settlement and lateral deformation of ballast layers. The present results further show that this confinement mechanism remains effective when the ballast voids are filled with foamed polyurethane.

However, unlike studies in which the geogrid was in direct contact with unbound ballast particles, the reinforcement mechanism in the present specimens did not depend solely on direct particle–geogrid interlocking. The geogrid was bonded to the polyurethane matrix, and the confinement force was transferred through the polyurethane before being transmitted to the ballast skeleton. This difference explains why the elastic modulus and Poisson’s ratio of the standard and geogrid-reinforced specimens remained nearly identical during the initial linear elastic stage. At relatively small strains, the deformation was insufficient to mobilize significant tensile resistance in the geogrid. As lateral expansion increased, the geogrid gradually developed tensile resistance and constrained the outward migration of ballast particles, thereby delaying crack propagation and increasing the ultimate compressive strength.

The observed behavior also agrees with the findings of Lee et al. [16] and Liu et al. [20], who indicated that the compressive response of polyurethane-bound aggregates is governed jointly by the polyurethane matrix and the aggregate skeleton. In the present study, the polyurethane primarily maintained specimen integrity and transferred stress during the early loading stage, whereas the geogrid became increasingly active after substantial lateral deformation developed. Therefore, the contribution of the geogrid was more pronounced in the nonlinear and failure stages than in the initial elastic stage.

### 5.2. Shear Behavior and Directional Dependence

The oblique shear tests showed that both geogrid-reinforced specimens exhibited higher peak loads than the standard PSB specimen. This result is consistent with previous findings that polyurethane bonding and geogrid reinforcement can improve the shear resistance of ballast by restricting particle rearrangement and suppressing particle extrusion [17,23]. Gundavaram and Hussaini [17] reported that polyurethane-bonded ballast exhibited greater shear strength and better resistance to particle degradation than conventional geogrid-reinforced ballast. The present study extends this observation by showing that geogrid confinement can provide an additional improvement even after the ballast particles have been bonded into a continuous polyurethane–aggregate composite.

A notable finding is that the shear resistance depended on the orientation of the shear plane. The peak load was higher when the shear plane corresponded to the unreinforced surface than when it corresponded to the geogrid-reinforced surface. This directional dependence can be attributed to the different stress states mobilized in the geogrid. When the top surface was used as the shear plane, the lateral expansion of the specimen generated tensile forces in the longitudinal geogrid ribs, allowing the geogrid to use its principal tensile resistance effectively. In contrast, when the reinforced side was used as the shear plane, the geogrid ribs were subjected mainly to combined shear and bending, for which a flexible geogrid provides less resistance. Therefore, the reinforcement efficiency was governed not only by the presence of the geogrid but also by the relationship between the geogrid orientation, the shear direction, and the associated deformation mode.

Previous research has emphasized the effects of geogrid aperture shape, aperture-to-particle-size ratio, and installation position on ballast reinforcement [21,22,24]. The present results indicate that the loading direction relative to the geogrid arrangement is another important factor for polyurethane-solidified ballast. This factor should be considered when determining geogrid placement in trackbed regions subjected to complex combinations of vertical, lateral, and shear loads.

### 5.3. Combined Reinforcement Mechanism and Engineering Implications

The combined reinforcement mechanism observed in this study can be interpreted as a sequential interaction between polyurethane bonding and geogrid confinement. At small strains, the polyurethane matrix and ballast skeleton carry most of the external load, while the geogrid deforms together with the specimen and contributes little additional stiffness. At larger strains, cracks initiate at the polyurethane–ballast interfaces and ballast particles tend to migrate laterally. The geogrid is then progressively mobilized, limiting outward deformation, redistributing stress, and delaying the development of localized failure.

This mechanism differs from that of conventional unbound geogrid-reinforced ballast, in which reinforcement primarily arises from direct mechanical interlocking between particles and grid apertures. It also differs from polyurethane-only stabilization, in which the load-bearing capacity depends mainly on the bonding strength and integrity of the polyurethane–aggregate interfaces. The combined system therefore provides complementary rather than simply additive reinforcement: polyurethane maintains structural continuity, whereas the geogrid supplies additional confinement after significant deformation develops.

From an engineering perspective, the results suggest that geogrid reinforcement may be particularly beneficial in locations where polyurethane-solidified ballast is subjected to large lateral deformation or localized shear, such as track transitions, bridge approaches, tunnel entrances, and sections with unstable subgrades. Nevertheless, the directional shear response observed in this study indicates that the orientation and location of the geogrid should be selected according to the expected principal deformation and loading directions rather than being determined solely by construction convenience.

## 6. Conclusions

This study focuses on polyurethane-solidified ballast (PSB). By conducting uniaxial compression tests and oblique shear tests on specimens of both standard PSB and geogrid-reinforced PSB, the study investigates their mechanical properties and deformation behavior under loading. The main conclusions are as follows:

(1) The average compressive strength of the standard PSB specimens was 1145.8 kPa, with an average maximum lateral displacement of 26.53 mm. The average compressive strength of the geogrid-reinforced specimens was 1628.0 kPa, with an average maximum lateral displacement of 22.76 mm. Compared to the specimens without geogrid reinforcement, the strength increased by 42.1% and the displacement decreased by 14.2%. This indicates that geogrids are highly effective in enhancing the compressive strength of the specimens and can effectively constrain lateral deformation under axial loading.
(2) The elastic modulus and Poisson’s ratio of the standard specimens and the geogrid-reinforced specimens are nearly identical, indicating that the geogrid is unlikely to provide reinforcement or constrain deformation during the linear elastic stage of the specimens.
(3) Under shear loading, the two geogrid-reinforced specimens exhibited higher peak loads than the standard specimens. Furthermore, when the shear plane was the top surface (i.e., the side without geogrid reinforcement), the specimens demonstrated higher overall shear strength. Under shear loading, the stress characteristics of the geogrid also served to constrain lateral deformation of the specimens and enhance their overall strength.
(4) Using DIC technology to capture the deformation process of PSB specimens under loading, it was observed that the deformation of the specimens primarily resulted from the compression of the polyurethane cells and the movement of the ballast particles. Following uniaxial compression and oblique shear tests, the failure patterns of the specimens consistently involved tearing of the polyurethane and extrusion of the ballast particles. Furthermore, specimens subjected to oblique shear exhibited more pronounced tearing failure characteristics in the shear direction.
(5) The three-dimensional random aggregate specimen model, which accounts for gradation, effectively replicates the actual deformation of the specimen. It also provides insight into the displacement behavior of the internal ballast, revealing that it gradually shifts toward the outer edge of the specimen under vertical loading. Comparison with experimental results confirms the validity and accuracy of the model parameters.

Beyond the fundamental mechanisms discussed above, our findings carry significant implications for practical railway engineering. The observed correlation between lateral deformation and geogrid confinement suggests that in real-world applications, strict control of confinement is crucial to prevent premature failure. Although our experiments were conducted under laboratory conditions, the observed phenomenon has practical significance for the actual engineering.

This study investigates the mechanical properties of PSB; however, due to constraints such as the experimental cycle and economic costs, there are still several limitations. The conclusions obtained in this study are limited to 150 mm cubic specimens prepared with the specific ballast gradation, polyurethane dosage, polyurethane density, geogrid type, aperture size, and reinforcement arrangement adopted in the present tests. Moreover, the experiments were conducted under quasi-static monotonic loading at room temperature, and only a limited number of specimens were tested, particularly in the oblique shear tests. Therefore, the quantitative results should be interpreted within the above experimental and material boundaries and should not be directly generalized to full-scale track structures, different material configurations, cyclic train loading, or long-term environmental conditions. Future studies should include repeated and larger-scale tests, different polyurethane and geogrid parameters, cyclic and dynamic loading tests, environmental durability evaluations, and further numerical validation under shear and realistic track-loading conditions.

## Figures and Tables

**Table 1:** Ballast particle size distribution after reducing the scale.

Side Length of Sieve Hole (mm)	26.5	19.0	16.0	13.2	9.5
Percentage of sieved mass (%)	100	70	35	10	0

**Table 2:** The content of each particle size range of aggregate in a single specimen.

Size Range (mm)	9.5–13.2	13.2–16.0	16.0–19.0	19.0–26.5	Total Mass
Standard specimen (kg)	0.52	1.29	1.81	1.55	5.17
Geogrid-reinforced specimen (kg)	0.50	1.25	1.75	1.50	5.00

**Table 3:** Compressive strength of specimens.

Specimen Label	Peak Load (kN)	Compressive Strength (kPa)	Average Strength (kPa)
C-1	27.29	1212.9	1145.8
C-2	25.94	1152.9	
C-3	24.11	1071.6	
CG-1	32.83	1459.1	1628.0
CG-2	38.70	1720.0	
CG-3	38.36	1704.9	

**Table 4:** Elasticity moduli and Poisson’s ratios of specimens.

Specimen Label	Stress Increment (kPa)	Elasticity Modulus (MPa)	Poisson’s Ratio
C-1	287.71	11.51	0.28
C-2	250.65	10.03	0.29
C-3	236.84	9.47	0.26
CG-1	246.29	9.85	0.25
CG-2	246.93	9.88	0.27
CG-3	276.67	11.07	0.29

**Table 5:** Maximum lateral displacement of the specimens.

Specimen Label	Maximum Displacement (mm)	Average (mm)
C-1	27.43	26.53
C-2	25.28	
C-3	26.89	
CG-1	22.72	22.76
CG-2	23.49	
CG-3	22.07	

**Table 6:** Parameters of the ballast particle templates.

Shape	Number of Faces	Number of Edges	Number of Vertices
(a) Near spherical	18	27	11
(b) Quadrate-1	14	21	9
(c) Quadrate2	14	21	9
(d) Pyramidal	14	21	9
(e) Needle-shaped	22	33	13
(f) Flaky	22	33	13

## Data Availability

The original contributions presented in this study are included in the article. Further inquiries can be directed to the corresponding authors.
